# Key Genes Associated with Pyroptosis in Gout and Construction of a miRNA-mRNA Regulatory Network

**DOI:** 10.3390/cells11203269

**Published:** 2022-10-17

**Authors:** Bing Bai, Yezhou Liu, Azierguli Abudukerimu, Tingting Tian, Meiting Liang, Rui Li, Yuping Sun

**Affiliations:** 1 School of Basic Medical Sciences, Xinjiang Medical University, Urumqi 830017, China; 2 Department of Microbiology, School of Basic Medical Sciences, Xinjiang Medical University, Urumqi 830017, China; 3 Xinjiang Key Laboratory of Molecular Biology for Endemic Diseases, Xinjiang Medical University, Urumqi 830017, China

**Keywords:** gout, inflammation, microRNA, pyroptosis

## Abstract

This study aimed to analyze key hub genes related to pyroptosis in gout and construct a miRNA-mRNA regulatory network using bioinformatic tools to elucidate the pathogenesis of gout and offer novel ideas to develop targeted therapeutic strategies for gout. Methods: The GSE160170 dataset was downloaded from the GEO database. The expression data extracted from the dataset were used to screen for differentially expressed genes (DEGs), which intersected with pyroptosis-related genes. These DEGs were analyzed via Gene Ontology (GO) and Kyoto Encyclopedia of Genes and Genomes (KEGG) enrichment analyses, and a protein–protein interaction (PPI) network was constructed to identify pyroptosis-related hub DEGs. The relationship between upstream miRNAs and the hub genes was analyzed, miRNA-mRNA networks belonging to gout disease were constructed and samples from patients with gout were used for experimental verification. The CTDbase tool was used to analyze the identified hub genes and construct a molecular docking model. Results: A total of 943 DEGs (380 upregulated and 563 downregulated) were identified by analyzing the data of patients with early-stage gout and healthy control individuals in the GSE160170 dataset. DEGs and pyroptosis-related genes were intersected to obtain 17 pyroptosis-related DEGs associated with gout; of which, 12 were upregulated, and five were downregulated. The results of GO and KEGG analyses revealed that the DEGs were enriched in inflammatory and immune signaling pathways. Additionally, the DEGs were found to regulate inflammatory responses and were associated with apoptosis. *TNF, IL-1β, NLRP3, CXCL8, PTGS2, NFE2L2, CASP8*, and *CD274* were identified as key hub genes in the PPI network, and a miRNA-mRNA network was constructed, which had 16 edges. Experimental validation revealed that *PTGS2* and *NFE2L2* were significantly upregulated, and *CASP8* and *CD274* were significantly downregulated in gout. In addition, miR-128-3p, miR-16-5p, miR-155-5p, and miR-20a-5p (associated with the miRNA-mRNA regulatory network) were significantly downregulated in gout. Five potential therapeutic drugs with stable *PTGS2* binding were selected to develop a molecular docking model. Conclusion: A miRNA-mRNA potential regulatory network was constructed based on pyroptosis-related DEGs associated with gout. miR-16-5p, miR-128-3p, miR-20a-5p, and miR-155-5p can potentially influence pyroptosis and the occurrence and development of gout by affecting the expression of the *PTGS2*, *CASP8*, *NFE2L2*, and *CD274* genes. Screening of celecoxib and resveratrol and other targeted drugs with stable binding. The findings of this study offer valuable insights into the regulatory mechanisms of gout and may help to identify Biomarkers and develop targeted therapeutic strategies for gout.

## 1. Introduction

Dysfunction of purine metabolism results in gout, a metabolic disease. Gout occurs as a result of the deposition of monosodium urate (MSU) crystals in joints. MSU crystals are formed when plasma uric acid levels are chronically elevated (hyperuricemia, HUA) beyond the saturation threshold. Gout often occurs in adult and elderly individuals and is most common among men. It develops in approximately 1–4% of the global population, and these patients tend to be younger. The death rate of gout is projected to increase to 55% by 2060 [1,2]. Gout and HUA denote different stages of the same disease. Uric acid accumulation and excretion disorders significantly increase uric acid levels in the body. HUA results in the local formation of MSU [3], which is phagocytosed by macrophages. Under these conditions, cathepsin B is released, the NOD-like receptor family pyrin domain-containing protein 3 (*NLRP3*) inflammasome is activated, and the signaling cascade of inflammatory factors is enhanced [4]. Gout can be classified under the symptom manifestation category of HUA, which is related to the stage associated with the outbreak of an inflammatory reaction. The first metatarsophalangeal joint in the foot is typically the most common site of gout onset, as gout typically develops in the joints and surrounding tissues of the lower limbs [5]. The clinical symptoms of gout can be superficially characterized as swelling, redness, pain, heat, and dysfunction around the affected area. Patients experience immense pain, which greatly restricts the movement of the affected area [6]. and affects the quality of life.

Pyroptosis is a type of programmed cell death, which is also referred to as inflammatory necrosis. Like gout, activation of inflammasome formation is the central response in pyroptosis, and proteins related to the gasdermin family are also involved [7]. Pyroptosis is associated with the generation of an innate immune response, and is a self-regulatory cell-death mechanism. Pattern recognition receptors (PRRs) on cells recognize the pathogen-associated model patterns (PAMPs) of infectious pathogens and activate *NLRP3* and other inflammasome complexes, resulting in caspase-1 activation. The gasdermin D (*GSDMD*) protein is cleaved and activated by active caspase. Under these conditions, the N-terminal fragment of the protein oligomerizes on the cell membrane and aggregates into pores, resulting in the rupture of the cell membrane. Subsequently, cellular contents and inflammatory factors (primarily *IL-1β* and *IL-18*) are released, and inflammatory cells accumulate [8,9] to eliminate pathogenic microorganisms to protect the host body. However, a high degree of pyroptosis can cause pathological reactions such as diabetic nephropathy and atherosclerosis [10,11].

The mechanism associated with the development of gout is very similar to that of pyroptosis, and *NLRP3* plays a key role in both mechanisms. Zhang et al. [12] reported that *NLRP3* is one of the target genes of miR-223, and miR-223-3p negatively regulates *NLRP3* to inhibit inflammation and pyroptosis (induced by sodium urate crystals) in rats and fibroblasts. Studies have examined the role of microRNAs (miRNAs) in the development of inflammatory diseases [13,14]. miRNAs are noncoding RNA molecules composed of approximately 20 nucleotides [15], and are widely present in eukaryotes. They can regulate gene expression by binding to the 3′-noncoding region (3′-UTR), which results in mRNA degradation or inhibition of translation. miRNAs function as clinical markers in the diagnosis of diseases [16] and have good circulatory stability in blood or body fluids [17,18].

In this study, we identified key genes associated with gout and pyroptosis by analyzing data extracted from the Gene Expression Omnibus (GEO) database. GO and KEGG enrichment analyses were performed to examine potential signaling pathways associated with gout. Thereby, based on previous miRNA-mRNA regulatory studies, the target genes were predicted and a miRNA-mRNA network was constructed, which contained 16 edges. Clinical specimens were collected to analyze and compare the expression of hub genes. Drugs were predicted based on the expression data of hub genes, and a molecular docking model based on small molecule compounds and hub genes was established to understand the pathogenesis of gout. Preliminary results showed that celecoxib can be used as the first-line drug to effectively alleviate gout. The docking model may help to develop new strategies for targeted therapy of gout.

## 2. Materials and Methods

### 2.1. Microarray Data Acquisition

“GOUT” was used as the keyword to download data from the GSE160170 (public) dataset [19]. “Homo sapiens” was used as the filtering condition while analyzing data extracted from the GEO database (https://www.ncbi.nlm.nih.gov/; accessed on 26 February 2022). The dataset included 6 healthy individuals (data number: GSM4861833–GSM4861838) and 6 patients with primary gout (data number: GSM4861839–GSM4861844). The expression matrix used was the GPL21827 (HuGene1_0-st) Affymetrix human gene 1.0 ST array (transcript (gene) version])

### 2.2. Differentially Expressed Genes Associated with Pyroptosis in Gout

The GEO2R tool (http://www.ncbi.nlm.nih.gov/geo/geo2r/; accessed on 26 February 2022) was used to analyze and compare the expression profiles of two or more groups to identify differentially expressed genes (DEGs) [20]. The tool was also used to normalize the data extracted from the GSE160170 dataset. Genes with FDRs of <0.05 and log FC of >1, or <−1 were identified as DEGs. The GeneCards database (version 5.8, https://www.genecards.org/; accessed on 1 March 2022) was used to integrate genetic data (genomic, transcriptomic, and proteomic data) from approximately 125 web sources [21]. Pyroptosis-related genes were identified by analyzing the integrated data, and intersected with the DEGs to obtain DEGs related to pyroptosis.

### 2.3. Functional Enrichment Analysis

The DAVID software (https://david.ncifcrf.gov/home.jsp; accessed on 1 March 2022) was used for GO and KEGG enrichment analyses of pyroptosis-related DEGs [22]. The DEGs were characterized, and key pathways were examined. GO enrichment analysis includes three independent categories: biological process (BP), molecular function (MF), and cell components (CCs). KEGG pathway analysis is based on genomic, chemical, and systemic functional information and is used to predict the role of proteins in cellular processes. A *p*-value of <0.05 was considered significant for both GO and KEGG analyses.

### 2.4. Construction of Protein–Protein Interaction and miRNA-mRNA Networks

A protein–protein interaction (PPI) network was constructed using the STRING database (http://string-db.org; accessed on 9 March 2022), with an interaction score of 0.4 [23]. Cytoscape was used to optimize and visualize the PPI network, and CytoHubba was used to identify important hub genes. The final hub genes were identified by intersecting the results obtained using the Degree, Maximal Clique centrality (MCC), and Maximum Neighborhood Component (MNC) algorithms. The NetworkAnalyst tool (https://www.networkanalyst.ca/; accessed on 19 March 2022) [24] was used to identify the miRNAs of pyroptosis-related hub genes and establish a miRNA-mRNA network.

### 2.5. Patient Selection

A total of 5 patients with gout receiving treatment at the Xinjiang Uygur Autonomous Region Hospital of Traditional Chinese Medicine and 5 healthy individuals (all men) were selected. The diagnosis of gout was based on the 2015 American College of Rheumatology/European League Against Rheumatism Collaborative Initiative gout classification criteria [25]. Patients with tumors, abnormal liver and kidney function, acute and chronic infectious diseases, diabetes mellitus, and hypertensive disorders were excluded. Blood samples were obtained from patients during the attack stages of gout. The study was approved by the Ethics Committee of Xinjiang Medical University and was performed in accordance with the ethical guidelines of the 1975 Declaration of Helsinki. The ethical review approval date for this experiment is 26 October 2020. The ethical approval code is K202010-12.

### 2.6. Quantitative Real-Time Polymerase Chain Reaction (qRT-PCR)

Total RNA was extracted from whole blood samples of patients using a total RNA extraction reagent (Solarbio, Beijing, China). The miRNA and mRNA reverse transcription reagents and PCR kits (Tiangen and Transgen Biotech, Beijing, China) were used according to the manufacturer’s instructions. The synthesized cDNA was amplified via real-time polymerase chain reaction (qPCR) (ABI Q6, Applied Biosystems Inc., Waltham, MA, USA). The primers used for PCR are listed in Table 1. GAPDH was used as an internal references, and the relative expressions of mRNAs and miRNAs were calculated using the 2^−ΔΔCt^ method.

### 2.7. Statistical Analysis

All data were expressed as mean ± standard deviation. Clinical data were analyzed using the SPSS software (version 28.0) (IBM, Armonk, NY, USA). The Student’s *t*-test was used to compare gene expression between groups (statistical significance: *p* < 0.05). Statistical analyses were performed using the GraphPad Prism 8 software. Pearson correlation coefficients were estimated for correlation analysis.

### 2.8. Drug-Gene Interaction and Molecular Docking Analysis

The CTD database (https://ctdbase.org/; accessed on 12 July 2022) [26] was used for predicting target drugs. The structure of the ligand molecule was downloaded from the PubChem database. The energy of the ligand molecule was minimized using Chem3D software and exported to mol2 format. Moreover, the PDB database to obtain the molecular structure of the target protein (PDB ID:5F19) [27,28]. The mol2 format of the small molecule and the PDB file format of the receptor protein were converted to PDBqt format and the active pocket was searched by using AutoDock tools 1.5.6 software. The search conformation range was set and the Vina script was run to perform docking simulations to obtain the docking energy [29]. Before molecular docking, water molecules and the ligand in the protein structure were removed, and hydrogen molecules and Gasteiger charges were added. A total of 10 docking poses were obtained for molecular docking calculations. The binding capacity was assessed using a semi-empirical scoring equation to select the most suitable dominant model in terms of geometry and energy, with the lowest binding free energy being the dominant conformation. The binding energy of ≤−7.0 kcal/mol indicated that the ligand molecule was strongly bound to the receptor protein. Finally, the ligand–receptor complexes generated via molecular docking were visualized in 3D using the PyMOL software (version 2.1) [30] to evaluate the biological reliability of the results.

## 3. Results

### 3.1. Expression of Pyroptosis-Related Differentially Expressed Genes

**Figure 1:**
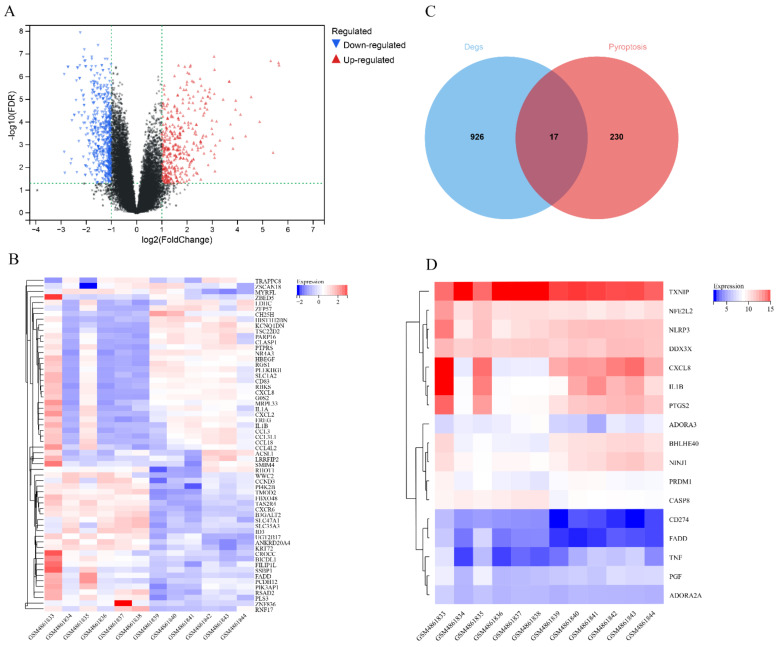
Identification of differentially expressed genes in gout. (**A**) Volcano map of differentially expressed genes associated with gout; (**B**) Heatmap demonstrating the top 30 upregulated and downregulated genes; (**C**) Venn diagram demonstrating the intersection between gout and pyroptosis-related differentially expressed genes; (**D**) Heatmap of 17 pyroptosis-related differentially expressed genes associated with gout.

The GSE160170 dataset was divided into the control (six healthy individuals) and experimental groups (six patients with gout). A total of 943 DEGs (380 upregulated and 563 downregulated genes) were identified by analyzing the expression data of both groups (Table S1). A volcano map was generated to visualize the DEGs (Figure 1A), and a heatmap was generated to visualize the top 30 upregulated and downregulated genes (Figure 1B). Additionally, 247 pyroptosis-related genes were identified using the GeneCards database (Table S2), and 17 pyroptosis-related DEGs associated with gout were identified from the intersection of the two groups of DEGs (Figure 1C,D, Table 2).

### 3.2. Functional Annotation of the Target DEGs

**Figure 2:**
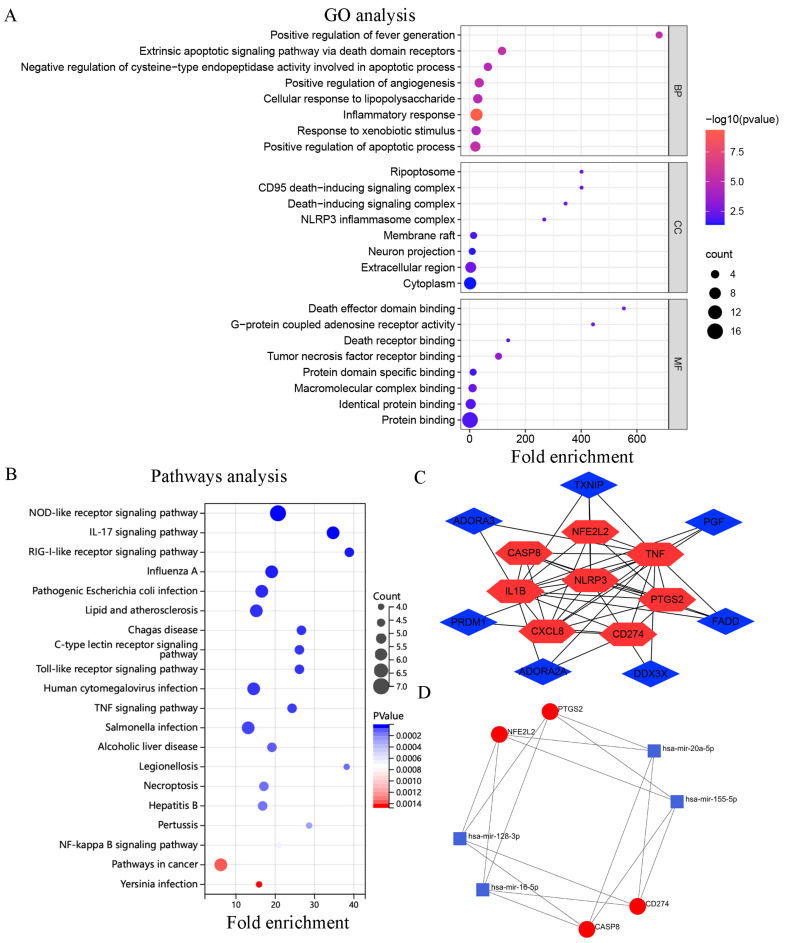
Enrichment analysis, PPI network, and miRNA-mRNA network of DEGs. (**A**) Top 8 GO terms; (**B**) Top 20 KEGG pathways; (**C**) PPI networks and hub genes; (**D**) MiRNA-mRNA network.

The results of GO and KEGG enrichment analyses are shown in (Table S3). GO analysis revealed that the DEGs were enriched in BPs such as inflammatory responses, positive regulation of apoptosis, and the extrinsic apoptotic-signaling pathway influenced by the action of death domain receptors. In addition, the DEGs were enriched in CCs such as the extracellular matrix, ripoptosome, and CD95 death-inducing signaling complex and MFs such as tumor necrosis factor receptor binding, death effector domain binding, and G-protein coupled adenosine receptor activity (Figure 2A). KEGG pathway enrichment analysis revealed that the DEGs were mainly involved in the RIG-I-like receptor, IL-17, and NOD-like receptor-signaling pathways, (Figure 2B).

### 3.3. PPI and miRNA-mRNA Networks

Unrelated genes (*NINJ1* and *BHLHE40*) in the PPI network were eliminated, resulting in the generation of a network with 15 nodes and 36 edges. Each node represented a protein, and each edge represented the interaction between proteins. A total of eight hub genes were eventually identified after the intersection of important hub genes identified via the MCC, Degree, and MNC algorithms (Table 3). Cytoscape was used for visualization (Figure 2C). On analyzing the hub genes, four mRNAs (*PTGS2*, *CASP8*, *NFE2L2*, and *CD274*) were found to be associated with 4 miRNAs (miR-155-5p, miR-128-3p, miR-16-5p, and miR-20a-5p). This finding is consistent with that of previous studies [31,32,33,34]. Subsequently, a miRNA–mRNA network was constructed, which contained 16 edges (Figure 2D).

### 3.4. General Information on the Study Population

No significant difference was observed in the average age of patients between the experimental and control groups (46.6 and 48.2 years, respectively) (*p* > 0.05). Serum uric acid levels were significantly higher in the experimental group than in the control group (*p* < 0.01) (Table 4).

### 3.5. Validation of Pyroptosis-Related Genes Associated with Gout

**Figure 3:**
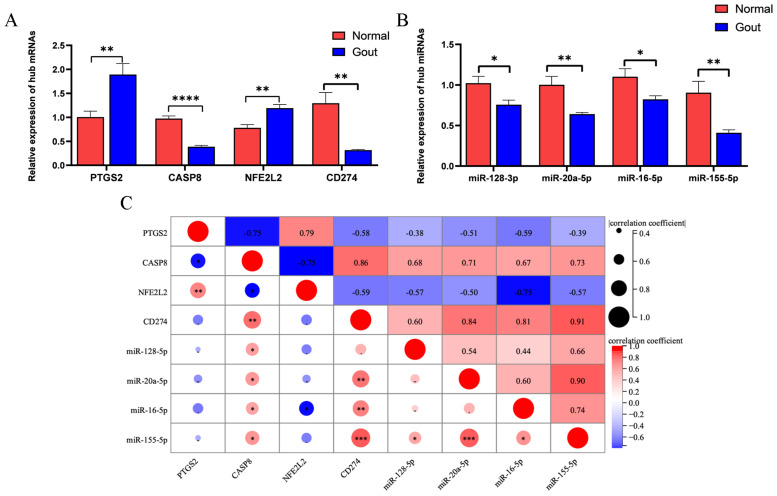
Verification of key differentially expressed genes related to gout and pyroptosis: (**A**) Expression of mRNAs; (**B**) Expression of miRNAs; (**C**) Pearson correlation analysis of mRNAs and miRNAs. (* *p* < 0.05; ** *p* < 0.01; *** *p* < 0.001; **** *p* < 0.0001).

qRT-PCR was used to analyze the predicted mRNAs and miRNAs. The mRNA expression of *PTGS2* and *NFE2L2* was significantly higher and that of *CASP8* and *CD274* was significantly lower in the experimental group than in the control group (Figure 3A). The results were consistent with those obtained via bioinformatic analysis. Additionally, miR-128-3p, miR-20a-5p, miR-16-5p, and miR-155-5p were downregulated in the experimental group (Figure 3B). Pearson correlation analysis revealed that a significant positive correlation between *CD274* and miR-155-5p (r = 0.91) and a significant negative correlation between *NFE2L2* and miR-16-5p (r = −0.75). These results indicated the relationship between the predicted miRNAs and mRNAs (Figure 3C).

### 3.6. Drug–Gene Interaction and Molecular Docking Analyses of PTGS2

**Figure 4:**
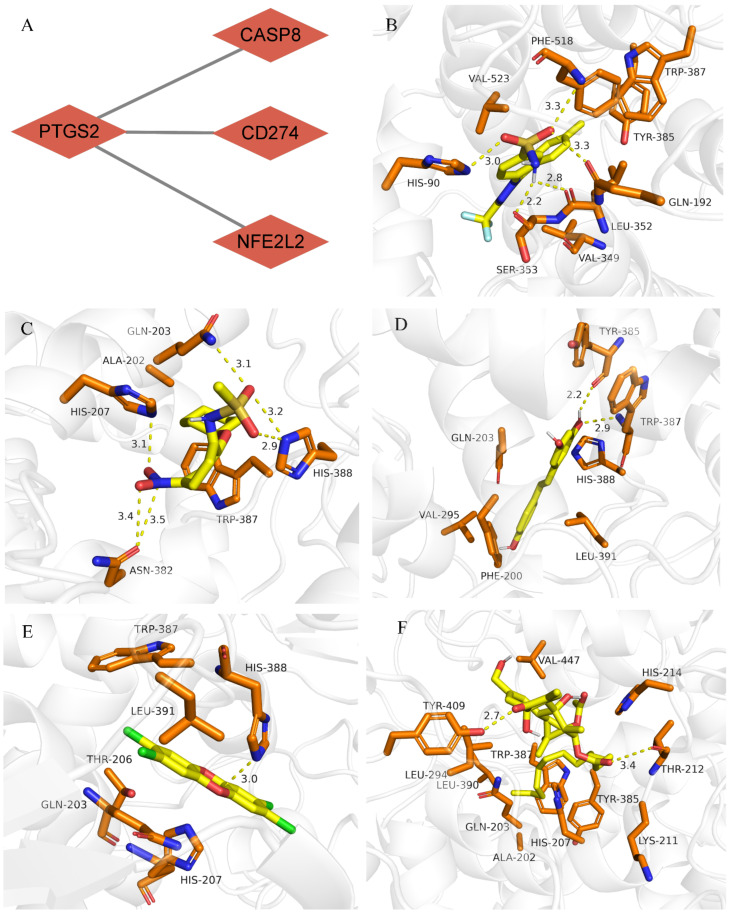
Molecular docking of the core gene *PTGS2* with drugs. (**A**) *PTGS2* was identified as the core gene; (**B**) Molecular docking of *PTGS2* and celecoxib; (**C**) Molecular docking of *PTGS2* and *N*-(2-cyclohexyloxy-4-nitrophenyl)methanesulfonamide; (**D**) Molecular docking of *PTGS2* and resveratrol; (**E**) Molecular docking of *PTGS2* and tetrachlorodibenzodioxin; (**F**) Molecular docking of *PTGS2* and tetradecanoylphorbol acetate.

*PTGS2* was the core gene among the four validated hub genes (Figure 4A). A total of 10 drugs that could bind to *PTGS2* with an interaction degree of >100 were selected from the CTD database (Supplementary Table S4). Of these 10 drugs, five small-molecule compounds were identified to have a strong binding affinity for *PTGS2* (binding energy ≤ 7 kcal mol^−1^; Table 5). Subsequently, the molecular binding sites corresponding to *PTGS2* and the five drugs were determined (Figure 4B–F).

## 4. Discussion

Gout manifests as severe and painful recurrent intermittent arthritis [5]. It is challenging to understand the pathogenesis of gout and identify therapeutic methods to effectively alleviate its symptoms and avoid its recurrence. The role of pyroptosis in gout has not been extensively studied. In this study, key genes associated with pyroptosis and gout were identified using bioinformatic tools. GO and KEGG analyses revealed that the genes were mainly associated with the generation of inflammatory responses, positive regulation of apoptosis, and inflammatory and immune-related signaling pathways. As the first line of defense against infection, the innate immune response is strengthened by PRRs. Toll-like receptors (TLRs), NOD-like receptors (NLRs), and RIG-I-like receptors (RLRs) [35] are important and extensively studied receptors. Innate immune cells recognize danger signals through membrane-bound receptors, namely, TLRs. These signals are transmitted to NLRs in the cytosol, which assembles inflammasomes. Inflammasomes activate caspase-1 and gasdermin-D via two synchronous mechanisms to initiate pyroptosis [36]. Pathways associated with pyroptosis and the regulation of TLRs, NLRs, and RLRs can potentially influence the generation of inflammatory responses in gout.

*PTGS2*, *CASP8*, *NFE2L2*, and *CD274* were identified as key pyroptosis-related genes associated with gout. These four mRNAs have been identified in previous studies on inflammatory diseases. Wei et al. [37] reported that miR-101-3p negatively regulates *PTGS2* and the proliferation and inflammation of fibroblast-like synoviocytes in rat models of rheumatoid arthritis. *CASP8* functions as a molecular switch during pyroptosis-mediated CD95 signaling [38]. Formation of the CD95 death-inducing signaling complex can result in apoptosis and autoimmune lymphoproliferative syndrome (ALPS) in humans and lymphoproliferative disease (LPR) in mice [39]. *NFE2L2* is a transcriptional activator that responds to oxidative stress [40] and promotes anti-inflammatory responses by coordinating with inflammatory cells. It also regulates gene expression by activating glutathione-s-transferase, which may help to alleviate osteoarthritis [40,41]. *CD274*, also known as *PD-L1*, is a member of the B7 family [42], and studies have demonstrated aberrant *CD274* signaling in animal models of acute inflammation [43,44].

To date, more than 2500 human miRNAs have been identified; however, the relationship between most miRNAs and mRNAs remains unclear [45]. In this study, we identified and analyzed key pyroptosis-related genes for miRNA prediction and verified the results using clinical specimens via qRT-PCR. The results revealed that the predicted miRNAs expression in the experimental group was significantly different from those in the control group. A few studies have examined the role of miRNAs underlying the occurrence of gout. Regulatory molecular mechanisms underlying the occurrence of gout can be elucidated by investigating the regulatory relationship between miRNAs and mRNAs, which may help in understanding the pathogenesis of gout and developing treatment strategies for it.

miRNAs are important regulators of various biological functions and influence the generation of various physiological immune responses. Chen et al. [46] reported that the balance between the synthesis and breakdown of the extracellular matrix (ECM) is disrupted during osteoarthritis. Stimulation of chondrocytes with interleukin-1β results in the downregulation of miR-128-3p, which in turn results in negative regulation of the overexpression of *WNT1*-inducible signaling pathway protein 1 (*WISP1*) and inhibits the proliferation of chondrocytes. The *NF-κB* pathway can induce apoptosis, pro-inflammatory cytokine production, and matrix degradation in chondrocytes. miR-20a is a member of the miR-17/92 cluster. In liver fibrosis, downregulation of miR-20A-5p can lead to the activation of transforming growth factor-beta (*TGF-β*) Induced by *TGF-β* receptor 2 (*TGFBR2*), which exacerbates inflammation [47]. miR-16-5p and miR-155-5p are associated with the generation of inflammatory responses in various cells [48,49,50]. To the best of our knowledge, this study is the first to report that miR-128-3p, miR-16-5p, miR-20a-5p, and miR-155-5p are downregulated in gout, Therefore, the findings of this study may help to understand the pathogenesis of gout and develop targeted therapeutic strategies for it.

At present, non-steroidal anti-inflammatory drugs and colchicine are primarily used for the treatment of gout [51,52]. These drugs exhibit anti-inflammatory, antipyretic, and analgesic properties and can alleviate the symptoms of gout. In this study, *PTGS2* was identified as an important target gene for drug screening. Small molecule compounds with a strong binding affinity for *PTGS2* were eventually used to develop a molecular docking model, which can be used to develop an efficient and reliable treatment strategy for gout. Among the screened drugs, resveratrol has been widely used to treat gout and exhibits good anti-inflammatory and antioxidant effects. Li [53] and Yang [54] et al. reported that resveratrol exerts therapeutic effects against gout by inhibiting TAK1 activity. Upregulation of *SIRT1* promotes MSU-induced autophagy and inhibits the generation of inflammatory response. Schumacher et al. [55] reported that celecoxib can alleviate pain and reduce the degree of inflammation in acute gout. Celecoxib is well tolerated by patients and does not exert negative effects on their health. In this study, the maximum extent of binding was observed between celecoxib and *PTGS2*, which is consistent with the findings of previous studies. However, celecoxib has been rarely studied, and further studies are required to validate the results of this study.

There are some limitations to this study. First, the study is a small cohort validation trial based on bioinformatics analysis. In vitro and in vivo experiments should be conducted in the future to understand the relationship between miRNA-mRNA, and a larger cohort should be analyzed. We identified key differential genes for gout associated with Pyroptosis, and a miRNA–mRNA network was established to predict the drug targets. We expect the treatment of gout to be simple, straightforward, and targeted. Therefore, we chose *PTGS2* as the target gene for our drug screen because it is the pivotal gene of the four genes. Targeting the drug to intervene in the pivotal gene may have unexpected effects on each gene in the network and to emphasise the importance of the gene network in the disease. However, we did not experimentally validate the results of molecular docking. In conclusion, this study can provide new perspectives on the pathogenesis of gout and new ideas on the screening of targeted drugs.

## Figures and Tables

**Table 1:** Fluorescent primers for qRT-PCR.

Gene	Primer Sequence (5′–3′)
*CASP8* (FORWARD)	GCCTTGATGTTATTCCAGAGAC
*CASP8* (REVERSE)	TCTGAAGTTCCCTTTCCATCTC
*PTGS2* (FORWARD)	ATCCTCCCACAGTCAAAGATAC
*PTGS2* (REVERSE)	CGCATACTCTGTTGTGTTCC
*CD274* (FORWARD)	TAGGAAGACGGGTTGAGAATC
*CD274* (REVERSE)	CACACTCACATGACAAGAAGAC
*NFE2L2* (FORWARD)	TCTCTTCTGTGCTGTCAAGG
*NFE2L2* (REVERSE)	AGCTCATACTCTTTCCGTCG
hsa-miR-128-3p (FORWARD)	CGTCACAGTGAACCGGTCTCT
hsa-miR-16-5p FORWARD)	GCTAGCAGCACGTAAATATTGGCG
hsa-miR-20a-5p (FORWARD)	GGGCTAAAGTGCTTATAGTGCAGGT
hsa-miR-155-5p (FORWARD)	CGCTTAATGCTAATCGTGATAGGGGT
*U6* (FORWARD)	GCTTCGGCAGCACATATACTAAAAT
*U6* (REVERSE)	CGCTTCACGAATTTGCGTGTCAT
*GAPDH* (FORWARD)	TGAGGCCGGTGCTGAGTATGT
*GAPDH* (REVERSE)	CAGTCTTCTGGGTGGCAGTGAT

**Table 2:** 17 Data on the differential genes associated with gout and pyroptosis.

Gene Symbol	logFC	Adj. *p*-Value	*p*-Value	Changes
*NLRP3*	1.45234415	0.0015	7.53 × 10^−5^	UP
*IL1B*	3.09126135	0.00366	0.00026	UP
*DDX3X*	1.80012202	0.0155	0.00184	UP
*PRDM1*	1.84476925	0.000238	5.77 × 10^−6^	UP
*NFE2L2*	1.73936101	0.00017	3.46 × 10^−6^	UP
*PGF*	1.03779492	5.53 × 10^−5^	6.64 × 10^−7^	UP
*PTGS2*	2.70238972	2.24 × 10^−5^	1.83 × 10^−7^	UP
*ADORA2A*	1.42834031	0.000129	2.36 × 10^−6^	UP
*TNF*	2.10325641	0.00709	0.00064	UP
*BHLHE40*	2.62218684	8.26 × 10^−6^	3.92 × 10^−8^	UP
*NINJ1*	1.72377655	0.00119	5.56 × 10^−5^	UP
*CXCL8*	5.62812965	2.40 × 10^−7^	1.18 × 10^−10^	UP
*CASP8*	−1.07127595	0.000251	6.19 × 10^−6^	DOWN
*TXNIP*	−1.04520555	0.00707	0.000637	DOWN
*CD274*	−1.59880911	0.00231	0.000138	DOWN
*ADORA3*	−1.36258112	0.0155	0.00185	DOWN
*FADD*	−2.26130412	1.20 × 10^−6^	2.24 × 10^−9^	DOWN

**Table 3:** Node score.

Gene	Degree Score	MCC Score	MNC Score
*TNF*	14	330	14
*IL1B*	12	326	12
*NLRP3*	9	290	9
*CXCL8*	8	270	8
*PTGS2*	7	264	7
*NFE2L2*	6	144	6
*CASP8*	6	144	6
*CD274*	6	32	6
*TXNIP*	4	24	4
*FADD*	4	24	4
*PGF*	3	6	3
*ADORA2A*	3	6	3
*DDX3X*	2	2	2
*PRDM1*	2	2	2
*ADORA3*	2	2	2

**Table 4:** Comparison of data of patients in two groups.

Mean ± Standard Deviation	Gout (*n* = 5)	Control (*n* = 5)	*p* Value
Age (years)	46.6 ± 6.43	48.2 ± 1.10	0.5981
Gender (male/female)	5/0	5/0	
Uric acid (mmol/L)	552.64 ± 76.82	341.8 ± 32.87	0.0005

**Table 5:** Drugs that interacted with PTGS2.

Corresponding Receptor Protein PTGS2	Binding Energy (kcal mol^−1^)
Celecoxib	−11
N-(2-cyclohexyloxy-4-nitrophenyl)methanesulfonamide	−7.8
Resveratrol	−8.1
Tetrachlorodibenzodioxin	−8.4
Tetradecanoylphorbol Acetate	−7

## Data Availability

Not applicable

## Supplementary Materials

The following supporting information can be downloaded at: https://www.mdpi.com/article/10.3390/cells11203269/s1, Table S1: 943 differential genes; Table S2: 247 pyroptosis genes; Table S3: Information about GO and KEGG; Table S4: Drugs with >100 predicted interactions.
